# A study on the hearing of children with non-syndromic cleft palate/lip

**DOI:** 10.1590/S1808-86942010000200004

**Published:** 2015-10-19

**Authors:** Maria Isabel Ramos do Amaral, José Eduardo Martins, Maria Francisca Colella dos Santos

**Affiliations:** Speech and hearing therapist. MSc student in Children and Adolescent health – Pediatrics Investigation Center/CIPED/FCM/UNICAMP; Otorhinolaryngologist- Otology and Cochlear Implant Department – ENT/Head and Neck Surgery Program – University of Campinas – UNICAMP; Speech and hearing therapist. PhD in Human Communication Disorders – UNIFESP/EPM, Professor and Coordinator of the Speech and Hearing Therapy Program – Medical Sciences School – University of Campinas – UNICAMP

**Keywords:** cleft palate, otitis media, hearing loss

## Abstract

Children with cleft lip/palate often present otitis media as a result of anatomic and/or functional alterations of the Eustachian tube.

**Aim:**

to analyze the results of Basic Audiologic Evaluation (BAE) and Auditory Processing Screening (APS) in children with cleft lip/palate.

**Study design:**

prospective cross-sectional cohort.

**Materials and methods:**

Forty-four male and female children, within the 8 to 14 age range with non-syndromic cleft lip/palate, referred by the institution where the study was carried out. The BAE was made up by an interview, otoscopy, threshold tonal audiometry, logoaudiometry and impedance test. The APS was made up of 3 basic tests: Sound Localization Test, Sequential Memory for verbal and non-verbal sounds and Dichotic Listening Test.

**Results:**

The BAE revealed that 77.27% of the children presented normal hearing; 13.6% had conductive hearing loss and 2.2% presented mixed hearing loss. 21.2% of the children had type C tympanometry curve; 7.1% had a type B curve and 3.5% had an Ad curve. The APS was altered in 72.7% of the children and 45.5% of them presented altered results on the Dichotic Listening Test.

**Conclusion:**

children with cleft lip/palate had altered results on BAE and APS, which justifies audiological and medical follow-up.

## INTRODUCTION

Hearing is the main link human beings have with the environment. In order to make communications possible, we need to first hear and understand, so that we can provide an answer using language.^1^ The first years of life have been considered paramount for language development and it is through hearing that the child comes in contact with the world of sound and language structures, which will later make up a structured communication system.^2^ Proper hearing system anatomical and physiological integrity, auditory pathway maturity and sound stimulation are essential for the acquisition and development of verbal language.

Hearing problems may represent an isolated clinical situation or be associated to other alterations. Among the alterations found associated with hearing, we emphasize cleft lip and palate (CLP). Congenital CLP develop during the embryonal and early fetal periods, clinically represented by no closure of the lip, palate or both.^3^ It is estimated that for every 1000 live births, one would have some type of cleft lip and/or palate.^4^

There are numerous systems used to classify and anatomically describe the type of fissure. The Spina classification^5^ is the one most used today, and it is based on lesion location in relation to the incisive foramen. The preincisive foramen cleft involves the lip and the alveolar arch and it can be uni or bilateral. The post-incisive-foramen cleft involves the hard and soft palate and can be uni or bilateral. The clefts which involve both the pre and the post foramen regions are called transforaminal.

Individuals with cleft lip and palate can have speech, dental, orthodontic and emotional problems. In children with CLP the most common hearing-related alteration is otitis media, caused by anatomical and/or functional malformations in the Eustachian Tube and in velopharyngeal sphincter (VPS) region. In order to better understand how this happens, it is necessary to better understand the structures involved.

The VPS is a muscle strip located between the oropharynx and the nasopharynx, involving the muscles of the soft palate, lateral and posterior pharyngeal walls, having a close relation with the Eustachian Tube, since the muscles are inserted in the tube cartilage and in the adjacent cranial base.^6^

The Eustachian tube is a tube which connects the tympanic cavity with the nasopharynx. During most of the time, the tube remains closed, and its main function is to balance the air pressure in the middle ear with that of the outside. It also protects against pressure and secretions coming from the nasopharynx and drains secretions produced in the middle ear.^7^ The soft palate stiffening muscle is responsible for opening the tube and it does so lowering the soft palate anteriorly during swallowing. For properly performing this task, it is essential to have an intact palate and its structures.

Thus, the main reason for having secretory otitis media in children with CLP is described in the literature as being chronic tube dysfunction, because of a failure in the tube opening mechanism. In patients with cleft palate, the tube does not open during swallowing because the soft palate stiffening muscle does not work properly, since it remains stuck in its palate insertion or it has some alteration in its course and insertion. If ventilation does not happen properly, functional tube obstruction can cause the presence of sterile fluid in the middle ear. Even when functionally obstructed, the tube can open and cause the aspiration of nasopharynx secretions, thus maintaining the secretory otitis media. This ear alteration is more frequent in incisive trans-foramen and post-foramen fissures, since these are the ones which involve the hard and soft palates^6^^,^^8^.

The presence of secretion in the middle ear or tympanic membrane perforation causes difficulties in sound transmission. The oscillating characteristic trait of recurrent otitis media causes fluctuation in sound detection and such situation causes lack of auditory stimulation consistency, difficulties in binaural integration and distortions in the received message – impairing hearing, speech and language development. Therefore, the hearing loss restricts the acoustic information organization and classification process - auditory processing. Such situation leads to a difficulty for the child to develop language concerning expression and understanding, and there can be problems associated with reading and writing (grapheme exchanges) and even behavioral difficulties or social distress.^9^

The early detection of the hearing loss is possible through Basic Auditory Evaluation tests (BAE), which pinpoint the degree and type of hearing loss, and they also report on the integrity of the entire peripheral auditory system. The identification of possible auditory processing disorders by means of the Auditory Processing Screening (APS) allows for a more proper treatment approach, besides better educating family members and health care professionals involved with the patient.

Thus, the goal of the present study was to evaluate the performance of children with non-syndromic cleft lip and palate and/or cleft palate alone regarding basic audiologic evaluation and auditory processing screening.

## MATERIALS AND METHODS

This study is a cross-sectional historical cohort, developed in the Health Care Area - CEPRE of the School of Medical Sciences (FCM) of the State University of Campinas, approved by the Ethics Committee of the FCM/UNICAMP, under protocol # 442/2005. We assessed 44 male and female children in the age range of 8 to 14 with non-syndromic cleft lip and palate and/or palate cleft, referred by the Brazilian Society of Craniofacial Rehabilitation Research and Care (Sobrapar). Such association provides medical care for patients with clefts and/or other dimorphic problems.

We took off the study those patients with other dimorphic problems associated with cleft lip and palate, with concomitant syndromes and/or those still not operated. The children were selected by Sobrapar, and all of those seen in the institution within the proposed age rage and who did not fit the exclusion criteria were called in by letter, telegram or a telephone call.

Although the result analysis was not carried out according to the type of cleft in each child, those children with cleft lip only were not included in the sample - this type of fissure does not affect VPS and could compromise the results from the study. The types of fissures presented by the sample are described on Table 1.

**Table 1:** Children with clefts, according to the type of cleft presented according to Spina (1972) considering uni or bilateral.

CLP Transforaminal*				Pre-foramen CP **		Post-foramen CP		Total			
Unilateral		Bilateral		Bilateral		Bilateral		Unilateral		Bilateral	
N	%	N	%	N	%	N	%	N	%	N	%
18	40,91	9	20,45	5	11,36	12	27,28	18	40,91	26	59,09

* CLP = Cleft Lip and Palate

** FP= Cleft Palate

After the parents or guardians signed the Free and Informed Consent Form, the following procedures were carried out: Interview, Otoscopy, Threshold Tonal Audiometer, Logoaudiometry, Immittance tests and Auditory Processing Screening.

The interview led to data collection about the child's recurrent otitis media past and whether or not the child had a ventilation tube (VT), and also data regarding the child's global development and the main auditory complaints associated with school performance and learning. Otoscopy was carried out by the ENT physician in order to check for the presence of cerumen, tympanic membrane perforation and/or other possible alterations.

Basic Audiologic Assessment Tests were carried out in a sound-treated booth, under the protocols proposed by Munhoz et al., 2003.^10^ We used the Interacoustic AC-30 audiometer with TDH-39 headphone and Interacoustic AZ-7 immittance device, properly calibrated.

Classification as to the level of hearing loss; considering that hearing loss were below 25 dBHL can impair language acquisition, suggested by Northern and Downs^2^.

The remaining criteria used for normality were:

Values in SRPI from 88 to 100%.^11^

Maximum compliance peak around pressure 0 of the Pa, volume equivalent from 0.3 to 1.3ml and acoustic reflex between 70 and 90 dB above the hearing threshold for pure tone.^12^

APS tests employed were carried out according to the Brazilian standardization proposed by Pereira and Schochat^13^ and Colella-Santos^14^, knowing:

### Sound Location Test in Five Directions

This test aims at assessing the sound location ability. The stimulus used for this test was the rattle, with percussion in five directions, without any visual clue. The kid was instructed to point to the sound direction. The reference criteria considered normal is to be correct in four or six directions, as long as the right and left are correct.

### Sequential Memory Test for Verbal and Non-Verbal Sounds

This test aims at assessing memory auditory skills for sounds in sequence (temporal ordering). for verbal sounds we used the following syllables “pa”, “ta”, “ca” and “fa” pronounced in three different sounds, without visual clues. For the non-verbal sounds we used four sound objects (rattle, bell, coconut and agogo bell) presented in three different sequences. The reference criteria considered normal is to be correct in two or three of the sequences presented.

### Digits Dichotic Test

This test assessed the skill for background figure for verbal sounds by means of the binaural integration stage. For 8 year-olds a percentage equal to or above 85% for the right ear and 82% for the left ear were considered normal. For children aged 9 years of age or above, a percentage equal to or higher than 95% for both ears is considered normal.

### Statistics

In order to do descriptive analysis and data crossing, we used the MINITAB Statistics Software. We calculated the number of right answers for each child, according to gender and ear. The performance of the children assessed was classified as normal or altered in each test, and the hearing loss was classified as normal or altered in each test, and also in type and level. We also considered the general result of the BAE and APS, and the normal or altered classification was done after comparing all the parameters with the existing normality criteria.

In order to study the statistical differences and dependence among the variables we used the SPSS Statistical Software Version 15.0. The main test was the Person's Chi-Squared non-parametric test, and when necessary we used the Fisher's non-parametric test. The level of significance used was established on 0.05 or 5%.

## RESULTS

Subject ages varied between 8 and 14 years (mean of 10.2 and standard deviation 2.1), 25 males (57%) and 19 females (43%). Statistical analysis was carried out in order to check the independence between gender and age, and the children were broken down into two groups per age range: 8 to 11 years (group 1) and 12 to 14 years (group 2). The statistical analysis showed no difference between age range and gender, because of it the data was plotted in one single age range group (p= 0.576 - Person's chi-squared test). Graph one shows the data collected during the medical interview regarding the history of otitis media episodes in childhood, use of a ventilation tube and tympanic membrane perforation.

Among the children reported as ventilation tube users, only 2 (4.5%) had hearing loss.

Tables 2 and 3 show the type and degree of hearing loss and the results from the immittance test, respectively. The statistical analysis did not show statistically significant difference between the genders, considering the percentages of right answers per ear (p= 0.68- Person's Chi-Squared test).

**Table 2:** Children with clefts, according to the hearing loss type and degree classification, as to gender.

Classification										
	Normal		Very mild conductive		Mild Conductive		Moderate Mixed		Total	
	N	%	N	%	N	%	N	%	N	%
Female	13	68.4	3	15.78	2	10.5	1	5.26	19	43.2
Male	21	84	3	12	1	4	0	0	25	56.8
Total	34	77.27	6	13.63	3	6.2	1	5.26	44	100

**Table 3:** Male and female children with clefts, according to the results from the immittance tests in the right (RE) and left (LE) ears.

			Tympanometric curve			Acoustic Reflex	
		A	B	C	Ad	Presente	Ausente
Male	RE	15	2	6	2	15	10
	LE	16	1	7	1	15	10
Female	RE	16	0	2	0	9	10
	LE	11	3	3	0	9	10
Total		58 (68,2%)	6 (7.1%)	18 (21,2)	3 (3,5.%)	48 (54,5%)	40 (45,5%)

Right ear X Left ear p = 0.712368 (Fischer)

The results regarding the mean value of right answers obtained in percentages, considering the Sequential Memory Tests for Verbal Sounds, Sequential Memory Test for non-verbal tests and digits dichotic test, are depicted on Table 4.

**Table 4:** Male and female children with clefts, considered normal and altered as to the Auditory Processing Screening.

Female					Male				Total				Fisher
	Normal		Altered		Normal		Altered		Normal		Altered		Gender
	N	%	N	%	N	%	N	%	N	%	N	%	M X F'
Sound location	16	80	3	20	23	92	2	8	39	88,6	5	11,4	p = 0,6450
Verbal SM	16	80	3	20	23	92	2	8	39	88,6	5	11,4	p = 0,6951
Non-verbal SM	16	80	3	20	23	92	2	8	39	88,6	5	11,4	p =0,7143
Dichotic digits	11	46,7	8	53,3	13	52	12	48	24	54,5	20	45,5	p = 1,000

**Graph 2:**
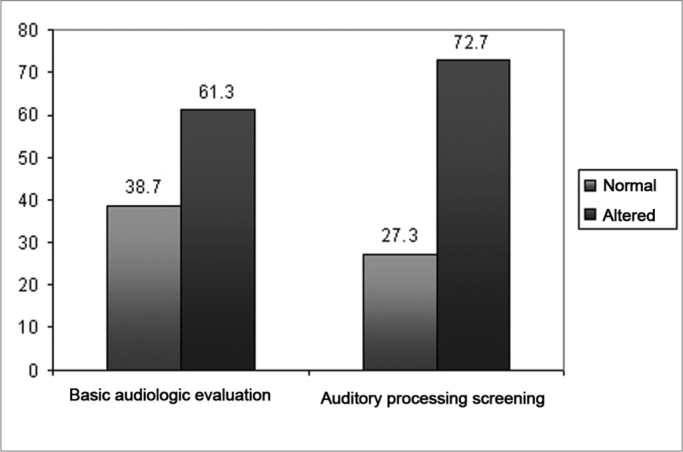
Distribution of the children considered normal and altered as to the Basic Audiologic Evaluation and Auditory Processing Screening.

The results regarding the entire distribution of the children considered normal and altered, as to the basic audiologic evaluation and Auditory Processing Screening, are depicted on Graph 2.

**Graph 3:**
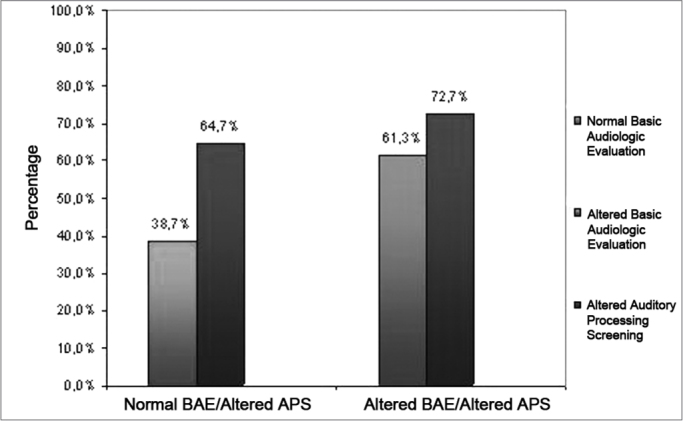
Children with clefts according to APS altered results in relation to BAE.

Graph 3 shows data cross over associated to the percentage of children who obtained altered results in the APS in relation to the results from the BAE (normal or altered).

**Chart 1:** Classification in relation to the degree of hearing loss in children, according to Northern and Downs, 2002.

Classification	Mean Hearing Loss (500, 1,2 and 3KHz)
Normal	0 – 15 dB
Very mild	16 – 25 dB
Mild	26 – 40dB
Moderate	41 – 70 dB
Severe	71 – 90 dB
Profound	Above 91 dB

## DISCUSSION

The data depicted on Table 1 shows the type of cleft presented by the children in the sample. Those children with cleft lip only were taken off the study. Studies report that functional and morphological alterations in individuals with CLP act as predisposing factors to a high occurrence of tube dysfunction, resulting in secretory otitis media and its complications;^15^^,^^16^ and the cleft involving only the lip and/or the dental arch, since there is no palate involvement, does not seem to interfere in the auditory sensibility of this population, having normal hearing.^17^, ^18^, ^19^

**Graph 1:**
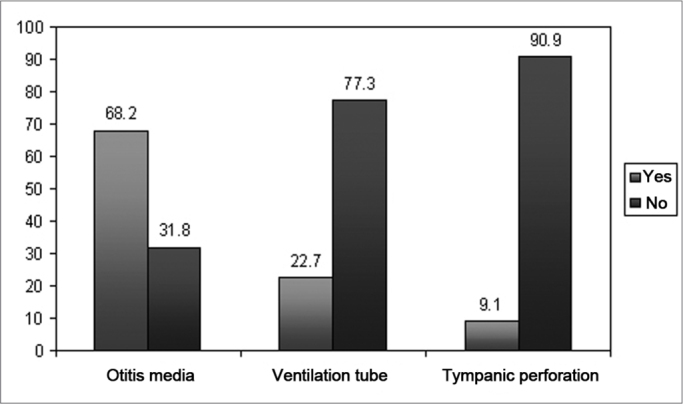
Children with clefts according to data collected from the interview with the parents.

According to the data collected during the interview held with the parents, we noticed that 68.2% of the children had a past of acute otitis media. Such data is in agreement with what is in the literature, pointing to a greater occurrence of repeated otitis media in the population with CLP, especially in the first years of life.^20^^,^^21^ Moreover, 22.7% of parents reported the use of a ventilation tube in the treatment of otitis media; and 9.1% reported that there was a tympanic perforation caused by frequent otitis media episodes (Graph 1).

In the literature studied, there is a major discussion regarding the use of early ventilation tube insertion in the treatment of otitis media in children with CLP. The early and intense use of a VT is advocated by some authors because of the major incidence of otitis media in this population, and the possible consequences that hearing loss can cause. Broen et al.^22^ reported on the importance of an early VT insertion in children from their study, since the later they ware inserted the worse were the auditory results found. More recently, Sheahan et al.^23^ stressed in their study the argument of some authors advocating a more conservative approach associated with VTs, based on the prevention of auditory alterations without an aggressive and early use.

In our study, the data collected during the interview, regarding 22.7% of the children having used VTs is relevant, since only 2 (4.5%) had hearing loss. Among the children who did not undergo the surgery, 8 (18.18%) had hearing loss. Therefore, there are reports that the VT was efficient in the treatment of hearing alterations caused by secretory otitis media in the children we studied, and it is in agreement with findings from other studies, which stresses the importance of VTs in assessment and treatment protocols for otologic alterations stemming from CLP.^24^ It is worth stressing that, according to the literature, there are possible sequelae after VT extrusion, such as otorrhea and/or the need for re-insertion. Therefore, it is necessary to properly follow up the children who are submitted to this procedure.^25^

As we analyze the results obtained from threshold tonal audiometry, we noticed that 34 of the children presented normal results (77.27%), 6 children (13.6%) had very mild conductive hearing loss, 3 children (6.8%) had mild hearing loss and one child (2.2%) had moderate mixed hearing loss (Table 2). The results found point to a greater incidence of conductive hearing loss in the CLP population and are in agreement with the findings in the literature we studied.

Chu and Mcpherson^26^ retrospectively studied 180 charts from Chinese children with CLP seen at the Cleft Lip And Palate Centre, Prince Philip Dental Hospital/University of Hong Kong. The results indicated that 13.4% of the patients had conductive hearing loss and 23.7% of the patients had altered tympanometric results. As it happened in our study, age and gender did not show significant relation with the altered results.

In another chart analysis study of 101 patients with CLP in the age between 8 and 25 years, carried out by Goudy et al.^3^, they noticed a higher conductive hearing loss incidence. Of the patients who had conductive hearing loss, 75% had the mild type, 21% moderate and only 4% had severe hearing loss of the mixed type. In our study we did not have any severe hearing loss, but we did have one child with moderate mixed hearing loss.

The results regarding tympanometric curve and Contralateral Acoustic Reflex were analyzed by ear (right and left), making up a total of 85 ears; and in 3 ears we could not do the test because of tympanic perforation. We noticed that 68.2% of the children had type A curve; 21.2% had type C curve; 7.1% had type B and 3.5% had a type Ad curve. The contralateral acoustic reflex was present in 54.5% of the children and 45.5% did not have this reflex (Table 3).

The type C tympanometric curve was the most frequent alteration found. It is characterized by a peak of maximum admittance, shifted for negative pressure, matching auditory tube dysfunction. Numerous studies associated the high incidence of auditory tube dysfunction with CLP patients as well as associating to it the main cause of secretory otitis media in these children, since the anatomical and/or functional conditions here are altered and favor permanent inflammation and build up of sterile fluid in the tympanic cavity.^20^^,^^27^^,^^28^ The type B tympanometric curve indicates the presence of fluid in the middle ear, consequence of inflammation and the presence of secretory otitis media. Very few studies were found in the literature regarding the incidence of type Ad curves described as being associated with laxity of the tympanic-ossicular system - caused by recurrent cases of otitis media and/or ossicular chain disruption.^29^ In our study, the two children who had a type Ad curve had numerous otitis media episodes reported in their interviews.

Some studies were found focusing not only the auditory system in children with CLP, but also the relationship of the hearing loss and language and learning disorders. Jocelyn et al.^30^ compared in their studies the results from speech and language skill evaluations with speech and hearing conditions of 16 children with CLP and children from a control group. These authors concluded that the children with the clefts had a lower index of cognitive and language development, in our study, we were not out to assess these skills. Nonetheless, through APS we could stress the influences a hearing loss, even if mild, can have in sound processing, directly associated to the learning process, school performance and social skills of these children.

The alterations involving Hearing Processing (HP) are associated to the mode with which the individual does not receive, but analyzes and interprets sounds by means of the auditory system structures. HP is characterized by a series of processes which succeed in time and allow the individual to perform sound metacognition and acoustic analysis. HP is mainly associated with the central nervous system and the cerebral cortex and is associated to the skills involved in decoding, organizing and coding the auditory sensorial information. Such skills depend on the innate biological capacity, intactness of the peripheral and central auditory system and the acoustic experiences in the environment.^31^ The results associated with the APS are depicted on Table 4 and Graph 2.

Regarding the Five Directions Sound Location Test employed, 11.4% (5/44) of the children had alterations, and 88.6% (39/44) had normal results. This test assesses sound location skills by means of a sound source discrimination auditory mechanism.

Regarding the Sequential Memory Tests for Verbal and Non-Verbal sounds, 11.4% (5/44) of the children had altered results in both tests, and 88.6% (39/44), had normal results. These tests assess temporal ordering ability regarding verbal and non-verbal sounds, by means of a sound discrimination auditory mechanism in sequence.

And, finally, regarding the Digits Dichotic Test, 45.5% (20/44) of the children had altered results for the age range; and 54.5% (24/44) had normal results. The Digits Dichotic Test has been recognized in the literature as important in the diagnosis of auditory processing alterations^32^ and is indicated as clinically proper in order to be used in an auditory processing screening, for being fast to apply and calculate results, easily understood by adults and children.^14^^,^^33^ This test assesses the figure-background skill for verbal sounds by means of the auditory mechanism of verbal sound recognition in dichotic hearing.

Analyzing the results in general, we can see that among the children with altered results in the BAE (61.3%), 72.7% of them presented altered APS. Moreover, children with normal BAE responses (38.7%) also had altered results in the APS (64.7%) (Graph 3). Based on these results, we stress in our study the importance of doing the Auditory Processing Assessment whenever possible in these children, considering their complaints, even if the results from the Basic Audiologic Assessment are normal.

Although we found very few studies associating CLP and auditory processing, there is evidence that children with CLP have a worse performance when compared to children without clefts. Belloni and Colella-Santos^34^, studied 25 children with CLP in the age range of 8 to 14 years, with the goal of analyzing the sample's auditory processing by means of Sound Location, Sequential Memory for Verbal and Non-Verbal Sounds tests and found alterations in 68% of the children in the sample. The study stressed the importance and need to have the Auditory Processing Screening being part of speech and hearing evaluation of children with CLP whenever possible and provide the proper subsidies which could guide treatment.

Cassab and Zorzetto (2002)^35^, used the auditory fusion test revised (AFT-R) in children with CLP, with the goal of investigating one of the central auditory processing skills - the temporal processing of the auditory system (temporal resolution) - in individuals with CLP, and compared these findings with the performance of children without CLP in the same age range, and found important alterations between the two groups, and the group of children with CLP presented worse results in relation to the control group. Of the 30 children from the CLP group we noticed that 22 had a past of otitis media in their first years of life, and 19 of them had altered performance in this test.

In a paper published by Lemos et al.,^36^, they reported the results from the Digits Dichotic Test (guided hearing stage) in children with CLP and compared them to the control group. They assessed 27 children with CLP and 25 children in the control group. The authors noticed that the group with CLP had correct answer percentages lower than the control group, for the right and left ears.

Having all the auditory involvement a child with CLP can have, we stress the importance of an otorhinolaryngological and audiological follow up as soon and as complete as possible, involving central and peripheral hearing. Proper treatment for secretory otitis media depends not only on proper ENT diagnosis and approach, but also on periodic follow up. A late diagnosis and the consequent lack of proper treatment can cause acute complications of prolonged episodes of otitis media, hearing impairment and the consequent effects on the child's linguistic and cognitive development.^37^

It is important to stress that even mild and very mild hearing losses cause significant loss for the child in relation to language development, learning and school performance, since one loses acoustic clues, especially those associated with the vowel sounds. Thus, proper medical and audiological treatment will avoid the onset of irreversible central and peripheral hearing damage in children with CLP, which can affect the development of oral and written language, causing problems in learning, school performance and social life.

## CONCLUSION

Based on the results from the our study we concluded that in this group of 44 children with CLP, the past of recurrent episodes of otitis media during infancy was present in most of the reports from parents, followed by the use of ventilation tubes and tympanic membrane perforation. Mild conductive hearing loss was the one more frequently found and the altered tympanometric curve more frequently found was the type C, suggesting tube dysfunction. Regarding APS, it was altered in children with audiometric alterations and also in children with normal peripheral hearing, and the Digits Dichotic Test was the one more frequently found altered. Thus, the CLP contributed to the occurrence of audiological problems and required proper ENT and audiological follow up.
